# Effects of brain microRNAs in cognitive trajectory and Alzheimer’s disease

**DOI:** 10.1007/s00401-024-02818-7

**Published:** 2024-10-30

**Authors:** Selina M. Vattathil, Sarah Sze Min Tan, Paul J. Kim, David A. Bennett, Julie A. Schneider, Aliza P. Wingo, Thomas S. Wingo

**Affiliations:** 1 Department of Neurology, University of California, Davis, 1651 Alhambra Blvd, Suite 200A, Sacramento, CA 95816 USA; 2 Department of Neurology, Emory University School of Medicine, Atlanta, GA USA; 3 Department of Psychiatry, Emory University School of Medicine, Atlanta, GA USA; 4 Rush Alzheimer’s Disease Center, Rush University Medical Center, Chicago, IL USA; 5 Department of Psychiatry, University of California, Davis, Sacramento, CA USA; 6 Veterans Affairs Northern California Health Care System, Sacramento, CA USA; 7 Alzheimer’s Disease Research Center, University of California, Davis, 1651 Alhambra Blvd, Suite 200A, Sacramento, CA 95816 USA

**Keywords:** Brain microRNA, Cognitive trajectory, Alzheimer’s disease, Beta-amyloid, Neurofibrillary tangles, Cognitive decline

## Abstract

microRNAs (miRNAs) have a broad influence on gene expression; however, we have limited insights into their contribution to rate of cognitive decline over time or Alzheimer’s disease (AD). Given this, we tested associations of 528 miRNAs with cognitive trajectory, AD hallmark pathologies, and AD clinical diagnosis using small RNA sequencing from the dorsolateral prefrontal cortex of 641 community-based donors. We found 311 miRNAs differentially expressed in AD or its endophenotypes after adjusting for technical and sociodemographic variables. Among these, 137 miRNAs remained differentially expressed after additionally adjusting for several co-occurring age-related cerebral pathologies, suggesting that some miRNAs are associated with the traits through co-occurring pathologies while others through mechanisms independent from pathologies. Pathway enrichment analysis of downstream targets of these differentially expressed miRNAs found enrichment in transcription, postsynaptic signalling, cellular senescence, and lipoproteins. In sex-stratified analyses, five miRNAs showed sex-biased differential expression for one or more AD endophenotypes, highlighting the role that sex has in AD. Lastly, we used Mendelian randomization to test whether the identified differentially expressed miRNAs contribute to the cause or are the consequence of the traits. Remarkably, 15 differentially expressed miRNAs had evidence consistent with a causal role, laying the groundwork for future mechanistic studies of miRNAs in AD and its endophenotypes.

**Supplementary Information:**

The online version contains supplementary material available at 10.1007/s00401-024-02818-7.

## Introduction

Understanding the diverse factors that contribute to late life cognitive decline is critical for developing strategies to mitigate its impact on individuals. Alzheimer’s disease (AD) is a neurodegenerative disorder defined pathologically by the accumulation of amyloid-β (Aβ) plaques and neurofibrillary tangles (NFTs) in the brain [18, 35] and is the leading primary cause of cognitive decline and dementia. AD pathology often co-occurs with multiple other age-related pathologies, which collectively account for about 40% of the variance in cognitive decline [7].

It is critical to note, however, that almost 60% of the variance in cognitive decline is not explained by common cerebral pathologies, highlighting the need to explore alternative risk factors. Thus, understanding molecular changes that are associated with AD and its hallmark pathologies after accounting for co-occurring cerebral pathologies offers insights into mechanisms of neurodegeneration and cognitive decline beyond those from pathologies. miRNAs are 22–25 nucleotide non-coding RNAs that suppress gene expression by promoting transcript degradation or inhibiting translation. One miRNA can influence expression of tens or hundreds of transcripts and thereby exert a widespread influence within a cell. While multiple studies have associated brain miRNAs with rate of cognitive decline [40], neurodegeneration [21, 31], beta-amyloid, and neurofibrillary tangles [30], the role of miRNAs in AD is relatively less characterized compared to brain transcripts and proteins. This is due to several factors, including small sample sizes, technological obstacles that limited the number of miRNAs profiled in a study, and heterogeneity across studies in profiling platforms and accounting for potential confounding factors. Recent advances in miRNA sequencing [19] allow us to expand the diversity of measured miRNAs at large scale to gain better insight into their role in cognitive trajectory, AD, and its hallmark neuropathologies using a large, well-characterized community-based cohort.

Here, we investigated the contribution of brain miRNAs to rate of cognitive decline over time, AD clinical diagnosis, and AD hallmark pathologies using brain miRNA sequencing from 604 participants with extensive antemortem and postmortem characterization. We addressed three specific hypotheses. First, we hypothesized that the abundance of certain miRNAs was associated with AD and its endophenotypes, and further posited that a subset of these miRNAs was associated with these traits through mechanisms independent of co-occurring age-related pathologies. To investigate this, we modeled the relationship between miRNAs and each trait using both minimally adjusted regression models (i.e., adjusting for sex, years of education, age at death, surrogate variables, and other technical variables) and fully adjusted regression models (including all above covariates plus up to 10 co-occurring age-related pathologies). While the minimally adjusted models identified a larger set of 311 differentially expressed (DE) miRNAs, the fully adjusted models identified 137 miRNAs whose association with the traits was independent of co-occurring cerebral pathologies. Second, we hypothesized that some miRNAs are associated with AD and its endophenotypes in a sex-dependent manner. We tested this using sex-stratified analyses, which identified five miRNAs with sex-biased differential expression for at least one AD endophenotype. Finally, we hypothesized that some miRNAs have a causal role in the pathogenesis of AD or its endophenotypes and tested this using Mendelian randomization (MR) analysis. The MR analysis identified 15 miRNAs that were not only differentially expressed but also had evidence consistent with a causal role in the pathogenesis of AD. Together, these findings lay the groundwork for future mechanistic and therapeutic studies to develop effective treatments for AD.

## Materials and methods

### ROS/MAP cohort

The Religious Orders Study (ROS) and Rush Memory and Aging Project (MAP), together referred to as ROS/MAP, are longitudinal clinical-pathologic community-based cohort studies focused on cognitive decline, dementia, and aging [3]. ROS recruits priests, monks, and nuns from across the United States, while MAP recruits lay people from retirement communities, social service agencies, and church groups in the greater Chicago area. Both studies enroll individuals without known dementia at baseline. ROS/MAP participants receive annual cognitive and clinical evaluations, and all participants are organ donors, provide informed consent, and sign an Anatomical Gift Act and a repository consent to allow their data and biospecimens to be repurposed. An Institutional Review Board of Rush University Medical Center approved the studies.

### Phenotype data

#### Clinical diagnoses

The final clinical diagnosis was made based on the recommendations of the National Institute of Neurological and Communicative Disorders and Stroke and the AD and Related Disorders Association by a neurologist using select clinical data but blinded to postmortem data [26]. It was treated as a binary outcome of cognitive impairment (MCI or Alzheimer’s dementia) versus no cognitive impairment (NCI), as reported in [2, 4, 5].

#### Cognitive trajectory

Cognitive trajectory is a person-specific rate of change of cognitive performance over time estimated for each of five cognitive domains and for global cognition [29]. Cognitive function in each domain (working memory, semantic memory, episodic memory, perceptual orientation, and perceptual speed) was measured at each timepoint using between 2 and 7 cognitive tests. Then, a single composite score at each timepoint was defined by converting the raw scores from the individual tests to Z-scores (using the mean and standard deviation of the cohort), and then averaging the Z-scores. Global cognition was summarized at each timepoint as the average of the Z scores from the full set of tests (19 total from the five domains). Cognitive trajectory for each domain and for global cognition was estimated using a linear mixed effects model with the longitudinal cognitive measure as the outcome. The models controlled for age at baseline, sex, and years of education. A positive trajectory value indicates improvement of cognitive performance over time, while a negative trajectory value indicates decline over time. The cognitive trajectories were treated as continuous outcomes.

#### Pathologies

Ten age-related pathologies were considered as outcomes and/or covariates. Beta-amyloid, neurofibrillary tangles, arteriolosclerosis, cerebral atherosclerosis, and cerebral amyloid angiopathy were treated as continuous variables, and Lewy body disease, TDP-43 pathology, gross infarcts, microinfarcts, and hippocampal sclerosis were treated as binary variables (absent v. present). Below, we briefly summarize each pathology measure.

The area occupied by amyloid beta protein was measured by immunohistochemistry and quantified by image analysis. The value is the percent area occupied by amyloid beta averaged over eight brain regions [39]. Neurofibrillary tangle pathology was measured as paired helical filament (PHF) tau density using immunohistochemistry and cortical density (per mm^2^) and was determined using systematic sampling. The value is the mean density averaged over eight brain regions [39]. For beta-amyloid and neurofibrillary tangles, the square root of the values was used to improve the normality of the variable distributions.

Arteriolosclerosis was graded by evaluation of the small vessels of the anterior basal ganglia for signs of deterioration or of arteriolar thickening resulting in narrowing of the vascular lumen [9]. Cerebral atherosclerosis was assessed by visual inspection of the vessels of the Circle of Willis, and severity was scored based on the number of affected arteries and the extent of involvement of each artery [1]. Cerebral amyloid angiopathy was described by assessing amyloid deposition using immunostaining for beta-amyloid in paraffin-embedded sections [8]. Arteriolosclerosis, cerebral atherosclerosis, and cerebral amyloid angiopathy were each summarized using a semi-quantitative score corresponding to four levels (none, mild, moderate, and severe).

Lewy body pathology was diagnosed based on algorithmic analysis and neuropathologist’s opinion of distribution of alpha-synuclein measured using immunohistochemistry. Lewy body disease was classified into four categories: not present, nigral-predominant, limbic-type, and neocortical-type [32]. TDP-43 pathology was measured using immunohistochemistry in six brain regions. TDP-43 pathology was coded as four stages: none, stage 1 (amygdala only), stage 2 (limbic [TDP-43 in hippocampus]), and stage 3 (neocortical) [28]. Presence of gross infarcts and microinfarcts was determined by pathologic assessment, blinded to clinical data, and reviewed by a board-certified neuropathologist [33, 34]. Gross infarcts included chronic, acute, and subacute infarcts and were detected by visual inspection of fixed slabs using the naked eye followed by histological confirmation via dissection. Presence of typical hippocampal sclerosis was assessed by severe neuronal loss and gliosis in CA1 and/or subiculum; evaluation was performed unilaterally in a coronal section of the mid-hippocampus at the level of the lateral geniculate body [27].

#### Sex

Female or male sex was based on self-report.

#### Race

Self-identified race was defined based on the participant’s response to the question ‘What is your race?’ with possible answers White; Black or African American; American Indian or Alaska Native; Native Hawaiian or Other Pacific Islander; Asian; or Other.

### MiRNA data

miRNA profiling has been described in detail previously [38]. Frozen post-mortem dorsolateral prefrontal cortex (dlPFC) samples were obtained from Rush University and 672 samples were selected for library preparation using New England Biolabs’ (NEB) NEBNext Multiplex Small RNA Library Prep Kit (NEB, Ipswich, MA, USA) and sequencing on an Illumina HiSeq 3000 (Illumina, San Diego, CA, USA) at the Emory Yerkes Genomics Core (Atlanta, GA). Sequencing depth ranged from 13.2 to 37.4 million reads per sample, with a median of 27.4 million reads per sample. Adaptor sequences were trimmed using Trimmomatic [6] (version 0.36), and miRNA counts were generated using mirDeep2 [13]. Reads were mapped to known human precursor and mature miRNA sequences obtained from MiRBase [20] (Release 22.1) and then filtered to only the 504 precursors and 857 mature miRNAs that have been included in the curated miRNA gene database MirGeneDB 2.1 [15] to minimize falsely detected miRNAs. Reads were allowed to map up to two nucleotides upstream of the mature sequence and up to five nucleotides downstream of the mature sequence, and with up to one mismatch. We removed reads for miRNAs with low abundance (< 1 RPM for > 50% of samples) and miRNA-precursor pairings absent from MirGeneDB 2.1. For miRNAs mapped to multiple precursors, we kept the entry with the highest total count across samples.

Sample outliers were identified using both raw counts and normalized counts. First, we iteratively removed samples that were greater than five standard deviations (SD) from the mean within the respective batch for total read count, trimmed read rate, and mapped read rate. We then filtered 11 samples from participants who did not self-identify their race as White. The analysis was limited to these participants because of small sample size for participants from other race groups. Next, the raw counts were normalized for library size and transformed to log2 counts using EdgeR. We then estimated the top 10 principal components (PCs) using the normalized data and removed 6 samples that were greater than 4 SD from the mean of either of the first two PCs. Finally, we used KING [24] with genotype data available from these samples to estimate and remove second degree and closer relatives. This final QC step filtered one sample. After quality control, data from 648 participants for 528 curated miRNAs present in the MirGeneDB database remained for analysis. Mapped read count for these samples ranged from 7.7 to 27.3 million reads per sample, with a median of 18.7 million reads per sample.

### RT-PCR validation

#### miRNA and sample selection

The validation experiments were focused on a subset of miRNAs and samples to maximize the value of the experiments with the available resources. From the putative causal miRNAs identified by SMR, we selected the three miRNAs associated with beta-amyloid or neurofibrillary tangles before adjusting for pathologies since these are outcomes of high interest. The selected miRNAs were miR-31-5p, miR-214-3p, and miR-3622a-3p. For selecting samples, we reasoned that we could increase the power of the analysis if, instead of randomly drawing individuals from the discovery sample, we used a case–control sampling scheme and selected samples in the extremes of the distributions for beta-amyloid and neurofibrillary tangles. Specifically, we ranked samples by their beta-amyloid and neurofibrillary tangles scores and got an averaged rank per sample, and then, contingent on tissue availability, selected samples with the lowest and highest ranks. We selected 45 cases (high beta-amyloid and high neurofibrillary tangles) and 45 controls (low beta-amyloid and low neurofibrillary tangles).

#### RNA extraction

RNA was extracted from dlPFC tissue samples using the Maxwell RSC simplyRNA Tissue Kit (Promega, catalog number AS1340). In detail, frozen brain tissue samples were dissected to 30 mg each. Each tissue sample was expeditiously homogenized in 200 uL of chilled 1-thioglycerol/homogenization solution using an ultrasonic homogenizer for 30–60 s, until complete homogenization indicated by no visible tissue fragments. If foaming occurred, sample was set on ice for up to 5 min for foam to settle. Homogenization solution was added to bring homogenate to a final volume of 200 μl, prior to addition to the cartridge.

Cartridges from the Maxwell RSC simplyRNA kit were loaded in in the deck trays of a Maxwell RSC 48 instrument, with well #1 facing away from the elution tubes. All sealing tape and any residual adhesive were then removed from the loaded cartridges. A sterile plunger was placed in well #8 of each cartridge, followed by the placement of an empty 0.5 mL elution tube into the elution tube position for each cartridge in the deck trays. 50 μl of nuclease-free water was pipetted to the bottom of each elution tube.

The sample lysates were loaded into well #1 followed by the addition of DNase I solution into well #4 of each cartridge. The Maxprep software was used to run the ‘simplyRNA Tissue’ method preloaded in the Maxwell RSC 48 instrument. Method-specific variables, such as the sample ID and elution volume, were entered into the system when prompted. Sample eluates were stored at  – 80 °C in the Maxwell elution tubes for downstream use.

#### Reverse-transcriptase PCR (RT-PCR)

RT-PCR was performed at the Emory Integrated Genomics Core. The 90 samples were randomized onto three plates by beta-amyloid, neurofibrillary tangles, sex, age, and RIN. All samples had RIN of at least 3.3, and median RIN was 6.7. Reverse transcription was performed using the TaqMan MicroRNA Reverse Transcription Kit (ThermoFisher, catalogue number 4366596) and qPCR reactions were performed using TaqMan MicroRNA Assays (ThermoFisher assay IDs 2279, 461768_mat, 464955_mat). Reactions were performed in triplicate for the three miRNAs of interest and U6 snRNA as a reference gene.

#### Data filtering

We first filtered outlier samples based on visual inspection of C_T_ plots. One sample with high C_T_ for the reference gene was dropped from all analyses, and one sample with high C_T_ for miR-214-3p was dropped from analyses for that miRNA only. We next filtered samples with failure of more than one out of the three triplicate reactions. This filter removed two samples from the analyses for miR-3622a-3p. Finally, we removed samples with C_T_ standard deviation (within triplicate) greater than 0.25. This filtered removed 8 samples for miR-214-3p, 15 samples for miR-31-5p, and 50 samples for miR-3622a-3p. The large standard deviation values for miR-3622a-3p are consistent with its generally lower abundance.

### Statistical analysis

All statistical analyses were done using R version 3.6.0.

#### Estimation of miRNA surrogate variables

miRNA surrogate variables (SVs) were estimated using the normalized log2 CPM abundance data with R package SVA. The miRNA SVs capture unmeasured sources of variation in the expression data, including cell type heterogeneity across samples, and are used as covariates in regression modeling to account for these potentially confounding factors. SVs were estimated for each model separately. The estimation produced 14 or 15 SVs to be included as covariates, depending on the model.

#### miRNA differential expression analysis

Differential expression was tested with voom-limma. Each of the nine outcomes was tested using two models, resulting in 18 models in total (Table 2). The first model for each outcome included batch, PMI, RIN, age at death, sex, education and miRNA surrogate variables as covariates. We refer to these models, which control only for technical and demographic covariates, as the minimally adjusted models. The second model for each outcome additionally included co-existing age-related pathologies as covariates. We refer to these as the fully adjusted models. Specifically, for the 6 cognitive trajectory models, ten pathologies (beta-amyloid, neurofibrillary tangles, arteriolosclerosis, cerebral atherosclerosis, cerebral amyloid angiopathy, Lewy body pathology, TDP-43, gross infarcts, microinfarcts, hippocampal sclerosis) were included as covariates in addition to the covariates included in the minimally adjusted models. For the beta-amyloid and neurofibrillary tangles models, the other nine pathologies were included as additional covariates. Finally, for the clinical diagnosis model, beta-amyloid and neurofibrillary tangles were included as additional covariates. The fully adjusted models were tested to identify miRNAs that are associated with each outcome independently of age-related pathologies.

False discovery rate (FDR) correction to control for multiple testing was performed using the Benjamini–Hochberg procedure and was applied to results of each model separately. In other words, FDR correction was based on the number of miRNAs tested. Differentially expressed miRNAs were defined as those with FDR < 0.05.

#### Pathway analysis

Pathway analysis was performed for each set of models (minimally adjusted and fully adjusted) in parallel using the predicted targets of the DE miRNAs for each trait. Predicted gene targets for each DE miRNA were extracted from TargetScan 8.0 [25] and low confidence predictions (cumulative weighted context++ score ≥ -0.2) were removed. A more negative TargetScan cumulative weighted context++ score indicates a stronger gene suppression effect between the miRNA and target gene. For each trait, we generated an initial list of genes by taking the union of the predicted target genes of the DE miRNAs. For the six cognitive trajectory traits, a single initial list was generated by taking the union of target genes across all six outcomes. This resulted in three lists of target genes for beta-amyloid, neurofibrillary tangles, and cognitive trajectories, respectively. The number of genes per list varied widely because the number of target genes is highly correlated with the number of DE miRNAs, which also differed across traits. Since the number of test genes substantially impacts pathway analysis results, we used a filtering procedure to select a comparable number of top target genes for each trait. First, we ranked the genes in each of the three lists by the number of DE miRNAs (for the relevant trait) that targeted them. We refer to the number of targeting DE miRNAs per gene as N_t_. We then counted the number of genes per list with N_t_ ≥ 1, N_t_ ≥ 2, and so on. Using these counts, we determined trait-specific cut-off values for N_t_ that would produce input lists per trait containing roughly the same number of genes. Pathway analysis was performed for each input list using DAVID [36] with the Reactome and WikiPathways databases in addition to the default databases. The background gene set was defined as the 15,582 genes present in a previously-created brain transcriptomic dataset generated from 637 post-mortem brains, which represented the brain-expressed genes that could be potentially targeted by brain miRNAs. Significance was defined as FDR < 0.05 using Benjamini–Hochberg adjustment.

#### Sex-biased differential expression analysis

Sex-specific differential expression analyses were conducted by splitting the dataset into male-only and female-only datasets and then applying the 18 models described previously except with the sex term removed from each model. The purpose of conducting the differential expression analyses in the sex-specific datasets was to improve sensitivity for miRNAs that are differentially expressed in only one sex but not the other, or that have opposite direction of effect in the two sexes. The significant associations from these sex-specific analyses were combined with the significant associations from the analysis using the ‘joint’ (males and females combined) dataset to make up the set of associations to test for sex-biased differential expression.

For each association of interest, we tested for sex-biased differential expression by adding a sex-by-trait interaction term to the relevant model and fitting this model in the joint dataset. Instead of Benjamini–Hochberg correction for multiple testing, we applied the stricter Bonferroni correction since there were fewer tests. The correction was applied to results of each model separately. Significant sex-biased differential expression was defined as sex interaction term Bonferroni p-value < 0.05.

#### Mendelian randomization (MR)

The summary data-based Mendelian randomization (SMR) and HEIDI tests [42] were used to integrate miRNA-QTL (miR-QTL) statistics with the SNP-trait association statistics to identify DE miRNAs whose association with the outcome trait is consistent with causality or pleiotropy. Only DE miRNAs with significant miR-QTLs within 500 Kb of the miRNA precursor were considered. The miR-QTL association statistics were estimated previously [38]. That analysis used the same miRNA data we used here for the miRNA differential analysis but was limited to a subset of 604 individuals based on the availability of both miRNA and genotype data. The association between SNPs and AD and its endophenotypes was estimated using PLINK2 using both minimally adjusted and fully adjusted models analogous to the miRNA differential expression analysis. The technical and demographic covariates for these models were age at death, sex, study, education, batch, PMI, RIN, and 10 genetic PCs. We defined miRNAs associated with the outcome trait through causality, pleiotropy, or linkage as those with SMR p-value < 0.05. Associations due to linkage were distinguished as those with HEIDI p-value < 0.05.

#### RT-qPCR Validation

For each sample for each miRNA of interest, the normalized expression value ΔC_T_ was calculated as the difference in C_T_ between the miRNA of interest and the reference gene. The association of case/control status (high beta-amyloid and high neurofibrillary tangles/low beta-amyloid and low neurofibrillary tangles) with miRNA expression was tested for each miRNA independently using linear regression with ΔC_T_ as the outcome, case/control status as the main predictor, and sex, age, education, PMI, RIN, and qPCR plate as covariates. Significant association was defined as p-value < 0.05.

## Results

### Demographics and correlation among traits

Demographic characteristics of the study sample are summarized in Table 1. Approximately 70% of the participants were female, and mean age at death was about 90 years. There was roughly equal representation of individuals with a final diagnosis of NCI, MCI, and Alzheimer’s dementia (AD).

**Table 1:** Characteristics of the ROS/MAP dataset

	*N*	Percent		
*Sex*				
Female	460	71		
Male	188	29		
*Clinical diagnosis at death*				
Normal cognition	234	36.1		
Mild cognitive impairment	187	28.9		
Alzheimer’s dementia	225	34.7		
Other dementia	2	0.3		

	Mean (SD)	Median	Range	Missing
Age at baseline visit	81.7 (6.7)	81.9	64.4–100.5	0
Age at death	90.1 (6.3)	90.7	71.2–108.3	0
Post-mortem interval (hours)	8.2 (5.1)	6.8	2.2–39.0	0
Education (years)	15.3 (3.3)	16	4.0–28.0	0

	Present	Percent	Missing	
Gross infarct (present)	278	43	0	
Micro infarct (present)	236	36	0	
Lewy Body disease (present)	141	22	23	
Hippocampal sclerosis	48	7	0	
TDP-43	323	50	5	

	Mean (SD)	Median	Range	Missing
Beta-amyloid	1.8 (1.2)	1.9	0.0–4.5	1
Neurofibrillary tangles	2.2 (1.2)	2	0.0–6.5	1
Cerebral atherosclerosis	1.1 (0.8)	1	0.0–3.0	1
Cerebral amyloid angiopathy	1.2 (0.9)	1	0.0–3.0	1
Arteriolosclerosis	1.1 (0.9)	1	0.0–3.0	2

	Mean (SD)	Median	Range	
Trajectory—global cognition	– 0.001 (0.087)	0.021	– 0.360 to 0.180	
Trajectory—working memory	– 0.003 (0.047)	0.005	– 0.224 to 0.105	
Trajectory—semantic memory	0.009 (0.089)	0.031	– 0.639 to 0.184	
Trajectory—episodic memory	0.000 (0.096)	0.016	– 0.317 to 0.222	
Trajectory—perceptual speed	– 0.007 (0.076)	0.003	– 0.300 to 0.213	
Trajectory—perceptual orientation	– 0.003 (0.039)	0.001	– 0.188 to 0.112	

Pathology measurements are described in Sect. Phenotype data. Briefly, beta-amyloid is represented by the percent area occupied by beta-amyloid averaged over eight brain regions. Neurofibrillary tangles are represented by the cortical paired helical filament (PHF) tau density (per mm^2^) averaged over eight brain regions. Cerebral atherosclerosis, cerebral amyloid angiopathy, and arteriolosclerosis are represented by semiquantitative variables with values 0–3 for none, mild, moderate, severe, respectively

To provide context for the relationships among AD endophenotypes and other age-related brain pathologies, we performed pairwise correlation analysis. The results illustrated a high degree of correlation between traditional measures of AD neuropathology (i.e., CERAD, Braak staging, beta-amyloid measured by immunohistochemistry [IHC], neurofibrillary tangles measured by IHC) and both AD clinical diagnosis and cognitive trajectory, consistent with previous results [7] (Supplementary Fig. 1). Seven of the eight measured non-hallmark AD cerebral pathologies (except microinfarct) were negatively associated with global cognitive trajectory, and three of the eight (TDP-43, Lewy bodies, and cerebral amyloid angiopathy) were positively associated with beta-amyloid and neurofibrillary tangles. As expected, cognitive trajectories were correlated with one another, with the Spearman correlation between global cognitive trajectory and each of the five domains ranging from 0.57 for perceptual orientation to 0.92 for episodic memory.

### MiRNAs differentially expressed in AD and its endophenotypes

Our first aim was to test miRNA differential expression with respect to AD and related endophenotypes. We considered nine AD-related traits: AD clinical diagnosis, beta-amyloid, neurofibrillary tangles, and cognitive trajectory for global cognitive function and five domains (episodic memory, semantic memory, working memory, perceptual speed, and perceptual orientation). The significant correlations between AD hallmark pathologies and other age-related pathologies motivated us to also test miRNA differential expression while adjusting for the effect of co-occurring age-related pathologies. To this end, we performed differential expression analysis for each trait using two regression models—a minimally adjusted model (adjusted for batch, age, sex, education, RIN, PMI, and surrogate variables) and a fully adjusted model (adjusting for the minimal covariates and up to 10 co-occurring pathologies, Table 2). Differential expression was tested for 528 miRNAs (Supplementary Table 1) that met the minimum abundance criteria in our dlPFC-derived small RNA sequencing data.

**Table 2:** Summary of covariates for minimally adjusted and fully adjusted models

	Covariates for minimally adjusted models	Covariates for fully adjusted models
Trajectory—global cognition	7 minimal covariates	7 minimal covariates + 10 pathologies
Trajectory—working memory	7 minimal covariates	7 minimal covariates + 10 pathologies
Trajectory—semantic memory	7 minimal covariates	7 minimal covariates + 10 pathologies
Trajectory—episodic memory	7 minimal covariates	7 minimal covariates + 10 pathologies
Trajectory—perceptual speed	7 minimal covariates	7 minimal covariates + 10 pathologies
Trajectory—perceptual orientation	7 minimal covariates	7 minimal covariates + 10 pathologies
Beta-amyloid	7 minimal covariates	7 minimal covariates + 9 other pathologies
Neurofibrillary tangles	7 minimal covariates	7 minimal covariates + 9 other pathologies
AD clinical diagnosis	7 minimal covariates	7 minimal covariates + beta-amyloid + NFT

The 7 minimal covariates are batch, PMI, RIN, sex, age, education, and surrogate variables

The 10 pathologies are beta-amyloid, neurofibrillary tangles, arteriolosclerosis, cerebral atherosclerosis, cerebral amyloid angiopathy, Lewy body disease, TDP-43, gross infarcts, microinfarcts, and hippocampal sclerosis

**Fig. 1:**
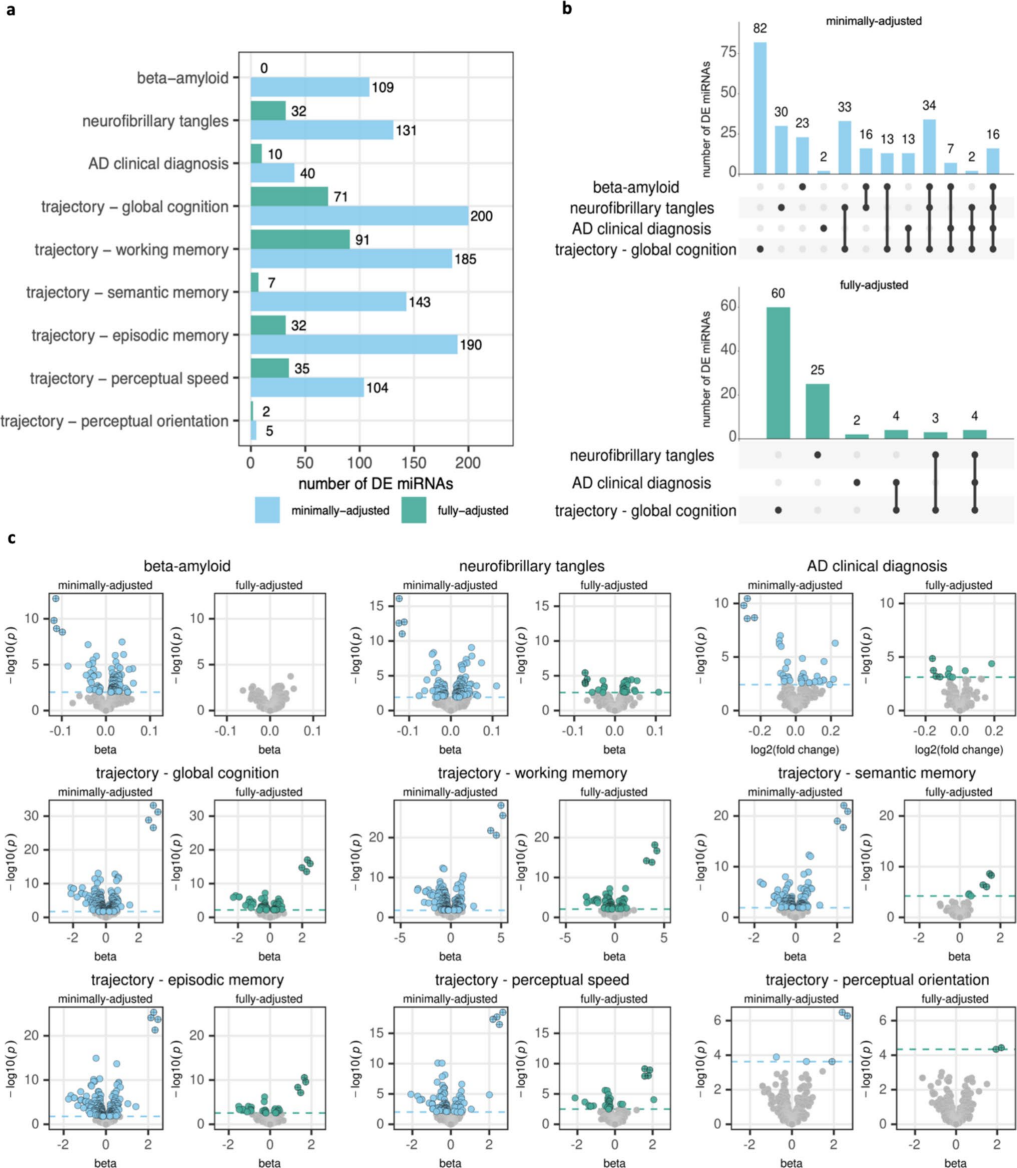
Summary of differentially expressed miRNAs from the minimally adjusted and fully adjusted models. See Table 2 for variables adjusted for in each model. **a** number of differentially expressed miRNAs per trait. **b** Upset plots for the number of DE miRNAs unique to or shared among the traits beta-amyloid, neurofibrillary tangles, AD clinical diagnosis, and trajectory of global cognition. The vertical bars show the number of DE miRNAs for the combination of traits indicated by the black circles in the matrix. Beta-amyloid had no DE miRNAs with the fully adjusted model and therefore is not included in the bottom panel. **c** Volcano plots for each trait. Dashed horizontal line indicates p-value corresponding to FDR < 5%. Points with gray fill indicate non-significant associations. Points with crosses indicate associations with miRNAs miR-132/212-3p/5p

With the minimally adjusted models, we found 1107 miRNA-trait associations (at FDR < 5%) across the nine traits, involving 311 unique miRNAs (Fig. 1a, Supplementary Tables 2, 3). Trajectory of global cognition had the highest number of DE miRNAs with 200, while trajectory of perceptual orientation had the fewest with only five DE miRNAs. As expected, given the positive correlation between AD clinical diagnosis, beta-amyloid, and neurofibrillary tangles, miRNAs associated with more than one of these traits have consistent direction of effect across the traits, regardless of the model used (Supplementary Tables 3 and 5).

With the fully adjusted models, we found 280 associations involving 137 unique miRNAs (Fig. 1a, Supplementary Table 4, 5). No miRNAs were associated with beta-amyloid after adjusting for neurofibrillary tangles. While a majority (843 of 1107, 76%) of the associations from the minimally-adjusted models were no longer significant after adjusting for pathologies, most (264 of 280) of the associations from the fully adjusted models were also detected using the minimally adjusted models. For the DE miRNAs significant using both models, the estimated effects all had the same direction using both models but were generally smaller in the fully adjusted models, consistent with attenuation of effect after adjusting for pathologies (Supplementary Table 6). Moreover, the DE miRNAs from the minimally adjusted models were more often associated with multiple traits than the DE miRNAs from the fully adjusted models (Fig. 1b), which is expected given that the fully adjusted models identify miRNAs that associate with the trait independently of co-occurring pathologies. In total, the differential analyses identified 317 DE miRNAs that originate from 269 miRNA precursors and 173 distinct miRNA clusters (set of miRNA precursors located within 10 Kb in the genome). Mature miRNAs originating from the same cluster are often regulated jointly, and we observed that miRNAs in a cluster frequently had positively correlated expression. miRNAs from the miR-132/212 cluster have the strongest associations for most traits (Fig. 1c).

To gain insight into how the 137 DE miRNAs from the fully adjusted models contribute to AD and its endophenotypes, we searched for enriched pathways among predicted target genes of the DE miRNAs (Supplementary Tables 7, 8, 9). For this analysis we considered the trajectory traits together, in addition to neurofibrillary tangles and clinical diagnosis. The traits had different numbers of DE miRNAs and therefore had different numbers of predicted targets. Specifically, the number of DE miRNAs for the trajectory traits, neurofibrillary tangles, and clinical diagnosis was 116, 32, and 10, respectively, and the number of predicted targets was 68,941, 18,836, and 5065, respectively. The size of the test gene lists strongly influences the pathway analysis results, so to make a fairer comparison we used trait-specific cut-offs on the number of DE miRNAs targeting each gene to select a comparable number (~ 1200–3900) of top targeted genes for each trait. For the cognitive trajectory traits, genes targeted by at least 10 DE miRNAs were enriched in zinc-finger proteins and lipoproteins and pathways involved in transcription, postsynaptic signalling, and cellular senescence. For neurofibrillary tangles, genes targeted by at least three DE miRNAs were enriched for lipoproteins and pathways related to transcription. Lipoproteins were also enriched among genes targeted by at least 1 DE miRNA for AD clinical diagnosis. For comparison, we also searched for enriched pathways among targets of DE miRNAs from the minimally adjusted models (Supplementary Tables 10, 11, 12) and found they were more numerous and heterogeneous than pathways from the fully adjusted models even when considering input lists of similar size by adjusting the gene rank threshold (see Methods). This is unsurprising given that we expect the DE miRNAs from the minimally adjusted models to have a less specific association with the outcomes. Consistently, the enriched pathways observed in the fully adjusted results were generally also enriched in the minimally adjusted results.

### MiRNAs with sex-biased differential expression for AD and its endophenotypes

**Fig. 2:**
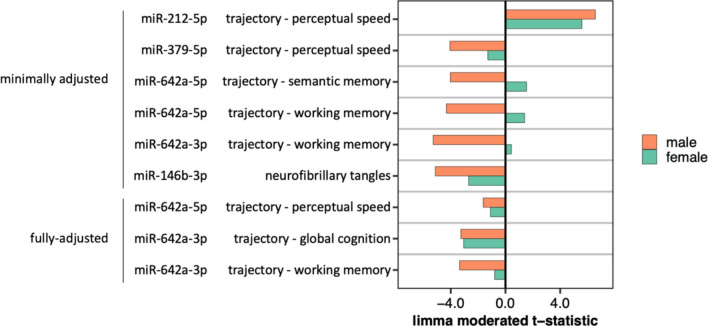
miRNA-trait pairs with significant sex-biased differential expression. The limma moderated t-statistic is the ratio of the beta estimate to the miRNA expression standard error after the standard errors have been moderated across miRNAs. The plot shows the moderated t-statistic from the male-only (orange) and female-only (green) analyses for each miRNA-trait pair (involving five unique miRNAs) with a significant sex interaction term, which allows comparison of the direction and magnitude of miRNA-trait association in each sex. Full results are in Supplementary Table 13

Since the prevalence of AD differs by sex, we hypothesized that some miRNAs would show sex-biased differential expression. To address this, we tested for sex-biased differential expression among miRNAs that were associated with AD and AD endophenotypes. We considered the 1387 associations described above, plus an additional 71 associations that were identified by applying the models in male-specific and female-specific datasets. Out of the 1458 associations, there were eight miRNA-trait pairs (five unique miRNAs and five unique traits) with evidence of sex-biased differential expression at Bonferroni-corrected p-value < 5% (Fig. 2; Supplementary Table 13). Comparing the miRNA-trait association moderated t-statistics in the male-specific and female-specific analyses, we noticed that the magnitude of effect was higher in males than females in each example. This is expected since the larger sample size for females gives us more power to detect smaller effects than we have in males. The effects were in the same direction in males and females for all the miRNAs except miR-642a-3p/5p. Specifically, these miRNAs were associated with trajectories of semantic and working memory in the minimally adjusted models with positive association in females and negative association in males. These miRNAs also had sex-biased differential expression in the fully adjusted models, but the effects are in the same direction between the sexes.

### Mendelian randomization

**Fig. 3:**
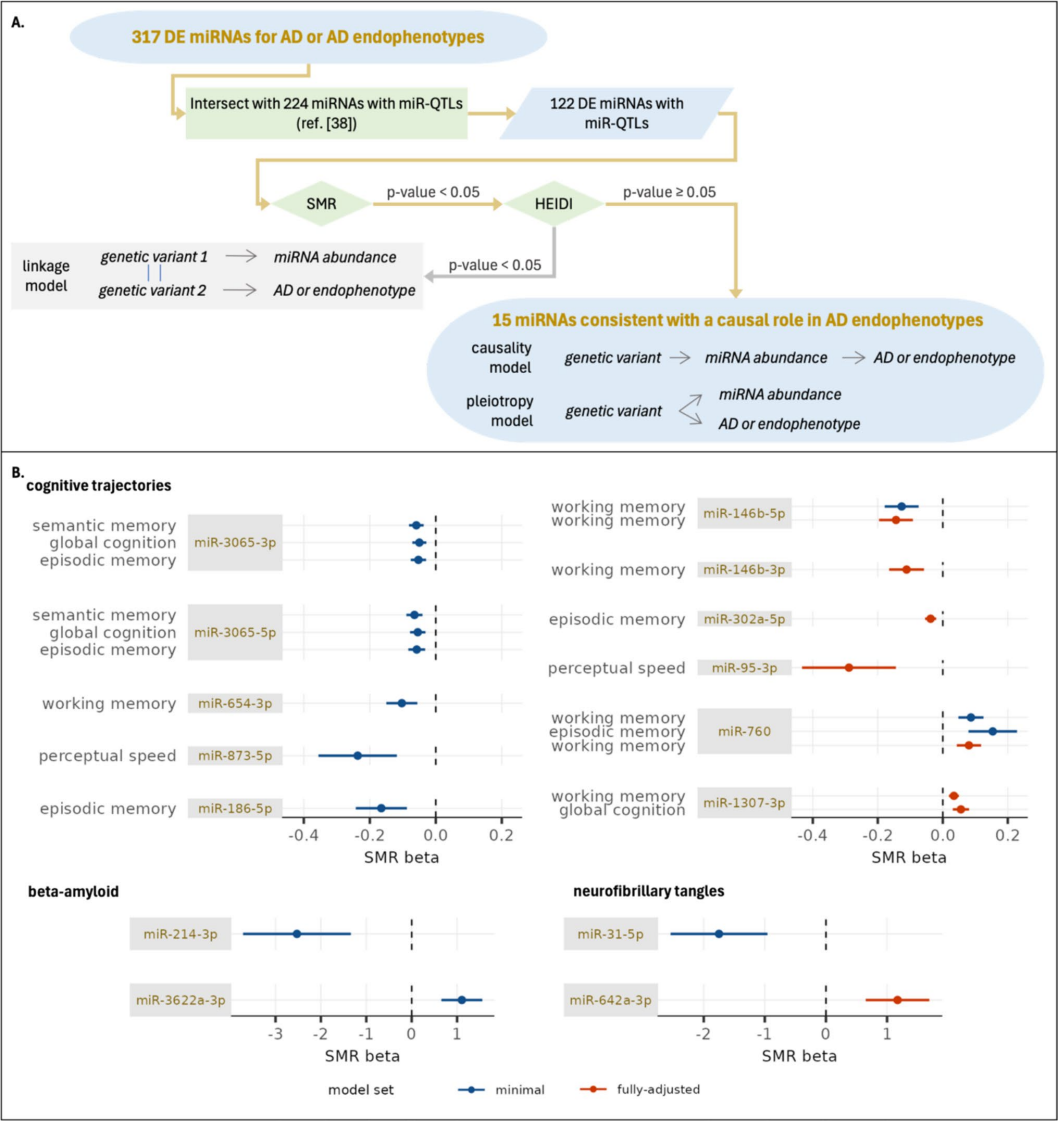
Identifying candidate causal miRNAs for AD and AD endophenotypes. A. Overview of workflow. The 122 DE miRNAs that had at least one miR-QTL in ref. [38] were tested using SMR and HEIDI. The SMR test identifies miRNAs associated with AD or AD endophenotypes through shared genetic association, which may occur through three models. The HEIDI test distinguishes the linkage model, in which the miRNA is associated with AD or AD endophenotype due to linkage of separate causal variants, from the causality and pleiotropy models, in which the miRNA is associated with AD or AD endophenotype due to a shared causal variant. The statistical tests do not distinguish causality and pleiotropy, so miRNAs consistent with either of these models are candidate causal miRNAs. B. Forest plots for 15 miRNAs consistent with a causal role in AD endophenotypes. Fifteen miRNAs passed the SMR/HEIDI thresholds and are candidate causal miRNAs. The SMR beta point estimates (filled circles) and 95% confidence intervals (solid lines) are plotted for candidate causal miRNAs for cognitive trajectories (top), beta-amyloid (bottom left), and neurofibrillary tangles (bottom right). None of the DE miRNAs for AD clinical diagnosis passed the SMR/HEIDI thresholds. Full results are presented in Supplementary Table 14

Observation of a DE miRNA suggests that the miRNA expression influences the development of AD and its endophenotypes or that the miRNA expression changes in response to AD and its endophenotypes. While both relationships are informative, we were interested in discerning the DE miRNAs that functionally contribute to AD or AD endophenotypes since these may provide insight into AD pathogenesis. To do so we used the summary data-based Mendelian randomization (SMR) and heterogeneity in dependent instrument (HEIDI) methods implemented in the software SMR [42]. The SMR workflow (Fig. 3A) tested whether DE miRNAs potentially mediate the effect of genetic variants on AD or its endophenotypes. This analysis incorporated SNP-miRNA association statistics that we estimated previously [38] using a sample set that overlapped the sample set used in the current manuscript. The SMR method requires a strong instrument variable, so we applied SMR for the 122 DE miRNAs with significant miRNA-associated SNPs (miR-QTLs) observed in the previous study. We identified 15 miRNAs that passed both the SMR and HEIDI tests, including two miRNAs for beta-amyloid, two for neurofibrillary tangles, and eleven for cognitive trajectory (Fig. 3B; Supplementary Table 14). The result for these miRNAs indicates that the SNP, miRNA expression, and AD endophenotype observations are consistent with a model in which the miRNA expression has a causal effect on the AD endophenotype. Alternatively, it is possible that the miRNA and outcome are pleiotropically affected by a shared causal SNP. In either case these 15 miRNAs are candidate causal miRNAs for cognitive trajectory and AD hallmark pathologies, making them exciting candidates for further mechanistic studies.

### RT-PCR validation

To further support the Mendelian randomization results, we sought to validate the association of miR-214, miR-31, and miR-3622a with amyloid and tangles using RT-PCR. These three miRNAs were putative causal miRNAs for beta-amyloid and neurofibrillary tangles, which are highly relevant to AD dementia and amenable to future testing using reporter assays. We sampled 45 participants with high beta-amyloid and high neurofibrillary tangles and 45 participants with low beta-amyloid and low neurofibrillary tangles (Supplementary Table 15) and measured miRNA abundance using RT-PCR. The ranking of median abundance for the three miRNAs based on RT-PCR was consistent with the observations from small RNAseq, with miR-31-5p having the highest abundance, followed by miR-214-3p and miR-3622a-3p. After QC filtering, the available sample size for differential expression analysis was 74 for miR-31-5p, 81 for miR-214-3p, and only 37 for miR-3622a-3p. The miR-3622a-3p sample set was small since many samples were excluded due to high C_T_ standard deviation, likely caused by the relatively low abundance of this miRNA.

We confirmed the positive association of miR-214-3p at p-value < 0.05 using a model that adjusted for PMI, RIN, plate, and sex, and the association remained significant when adjusting for age at death and education (Supplementary Table 16). We further observe positive association of miR-31-5p using a simplified model that did not adjust for sex (Supplementary Table 16). It is reasonable to consider the association without adjusting for sex since sex is highly associated with beta-amyloid and neurofibrillary tangles pathology but likely has minimal direct influence on miRNA expression. In sum, despite the limited power given the relatively small sample size, the technical validation experiments find robust support for the positive association of miR-214-3p with beta-amyloid and neurofibrillary tangles and suggestive support for positive association of miR-31-5p with both pathologies. Future validation studies with more power or in an independent sample would further add to the confidence of these findings.

## Discussion

Using small RNA sequencing from over 600 extensively characterized study participants, we identified differentially expressed miRNAs for AD, the hallmark AD pathologies beta-amyloid and neurofibrillary tangles, and multiple cognitive trajectory traits. We did this with both minimally adjusted models and models that included other co-occurring age-related pathologies (i.e., fully adjusted) to understand which miRNAs are associated with the trait of interest independently of age-related pathologies. The minimally adjusted models identified 311 miRNAs associated with beta-amyloid, neurofibrillary tangles, AD clinical diagnosis, and/or one of the six cognitive trajectories. The fully adjusted models identified 137 differentially expressed miRNAs for those traits, most of which were also found in the minimally adjusted analysis. These findings suggest that while many miRNAs may be associated with AD and rate of cognitive decline through their relationship with age-related pathologies, a substantial subset of miRNAs are at least partially involved through processes that are independent of these pathologies.

Excitingly, this is the first study to ask which DE miRNAs have a potential causal role in AD and its endophenotypes. We applied MR analysis to the DE miRNAs and identified 15 DE miRNAs whose associations with beta-amyloid, neurofibrillary tangles, or cognitive decline were consistent with a pleiotropic effect or a causal role of the miRNA. These results highlight that these miRNAs are not only biomarkers of these traits but also potentially influence their risk. These miRNAs are promising targets for further mechanistic studies. For example, our MR analysis found miR-146b to be potentially causal for trajectory of working memory, and recent experimental work found that miR-146b deficient mice had better episodic recognition memory and fear memory [10].

We tested whether sex modified the association between miRNA expression and AD endophenotypes. This inquiry was motivated by differences in prevalence in AD and cognitive decline by sex [12, 22, 23], and sufficient sample size to detect sex-biased miRNA expression. We found sex-biased differential expression of miR-212-5p, miR-379-5p, and miR-642a-3p/5p for trajectory traits and miR-146b-3p for neurofibrillary tangles, suggesting these miRNAs may be linked to the outcomes through sex-biased mechanisms. These results should be interpreted acknowledging that biologic sex, which was studied, is correlated with lifestyle, education, socioeconomics, employment, and survival and the results of sex-biased expression cannot be disentangled from those correlated factors in our results.

Brain miRNA differential expression analysis is a promising approach to uncover mechanisms underlying brain neurodegenerative and cognitive traits. However, existing studies for AD have produced inconsistent results, likely due to differences in study design, covariates, and small sample size (typically fewer than 85 individuals) [11, 37, 41]. Larger studies (sample size > 400) using NanoString data from ROS/MAP participants identified only a handful of differentially expressed miRNAs [30, 40], possibly due to NanoString’s lower detection sensitivity and dynamic range compared to miRNA sequencing and qPCR [17]. The recent study by Dobricic et al. [11], which analyzed brain miRNA expression in 190 individuals, was the largest study to date using qPCR or small RNA sequencing but focused on only six top miRNAs from a recent meta-analysis [37]. Of the 25 miRNAs from that meta-analysis, our analysis confirmed two DE miRNAs (miR-129-5p and miR-132-5p) for clinical diagnosis and 12 miRNAs for global cognitive trajectory and identified many novel associations. Importantly, we limited analysis to miRNAs validated through expert curation [15] instead of considering all entries in miRBase, which contains many false positive or non-miRNA small RNAs [14]. By doing so, we can be confident that the miRNAs we identified are reasonable candidates for follow-up [16].

We note some limitations of the study. First, the sample is imbalanced toward females, which is a common feature of participants who consent to organ donation during life. In the sex-specific analyses, the sex imbalance creates a bias toward detecting miRNAs with strong association in males. Future sex-specific analyses in a sample with balanced sex will be more powerful and less biased. Second, replication in an independent sample would increase the impact of these results. The large sample size, extensive pathology assessment, and sequencing-based approach for miRNA profiling in our study makes it impossible currently to find a comparable independent sample for replication.

In summary, this study expands the catalog of differentially expressed brain miRNAs for AD and AD endophenotypes, identifying miRNA independent of co-occurring pathologies, with sex-biased differential expression, and with potential causal links to AD. These findings support further research into the role of miRNAs in AD pathogenesis, particularly the 15 DE miRNAs with evidence of causal effects, which warrant investigation in mechanistic studies.

## Supplementary Information

Below is the link to the electronic supplementary material.

Supplementary file1 (XLSX 1287 KB)

Supplementary file2 (DOCX 235 KB)

## Data Availability

ROS/MAP resources including the miRNA data can be requested at https://www.radc.rush.edu. miRNA data will also be available on www.synapse.org at https://www.synapse.org/Synapse:syn51247298.

## Code availability

Summary-data-based Mendelian randomization and HEIDI were performed using SMR v1.03 (https://yanglab.westlake.edu.cn/software/smr/). All other analyses were performed in R version 3.6.0 using base functions and the following R packages: data.table v1.14.2 for data manipulation; edgeR v 3.28.1 for expression normalization; sva v.3.34.0 for surrogate variable analysis; voom-limma v3.42.2 for differential expression analysis; qvalue v2.15.0 to estimate qvalues; GenomicRanges v.1.38.0 for merging genomic intervals.
