# Supplementary material for: Effects of brain microRNAs in cognitive trajectory and Alzheimer’s disease
Source: Acta Neuropathol. 2024 Oct 30;148(1):59. doi: 10.1007/s00401-024-02818-7 (PMC11525270; doi:10.1007/s00401-024-02818-7)
Supplement: Supplementary file 2 — Supplementary file2 (DOCX 235 KB) [file 401_2024_2818_MOESM2_ESM.docx]

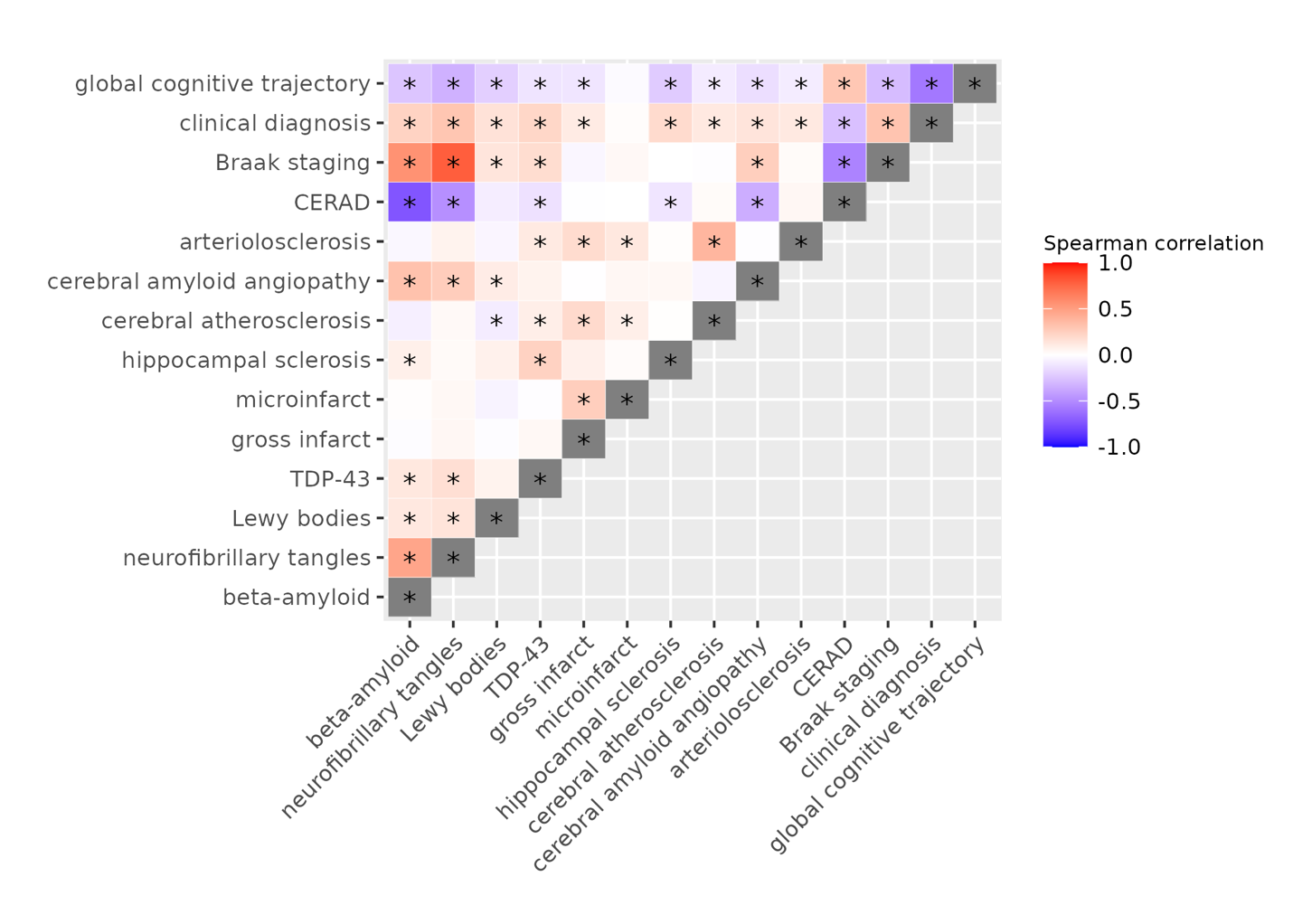


Supplementary Figure 1. Pairwise correlation among global cognitive trajectory, clinical diagnosis, and pathologies. Asterisks indicate Spearman p-value < 0.05. In our analysis we used beta-amyloid and neurofibrillary tangle measures as derived on the ROS/MAP participants, but we include Braak staging or CERAD to provide context for the outcomes.
